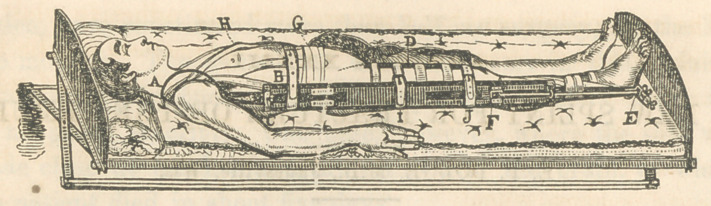# A New Splint for Fracture of the Femur

**Published:** 1862-10

**Authors:** E. F. Dodge

**Affiliations:** Janesville, Wis.


ARTICLE XXXIII.

### A NEW SPLINT FOR FRACTURE OF THE FEMUR.

E. F. DODGE, M.D., Janesville, Wis.

MESSRS. EDITORS OF THE EXAMINER:—During the course of lectures by Prof. ANDREWS of 1861-2, in the Medical Department of Lind University, he exhibited and explained the application of a rude splint, by which both extension and counter-extension could be made by the use of adhesive plaster in all cases of fracture of the femur and leg; doing away with the perineal band, so long used and revered by all of the disciples of Æsculapius, except Dr. GILBERT, who used the adhesive plasters in fractures of the femur and leg, but in such a manner as to amount to the same thing as the perineal band. (See description of GILBERT'S apparatus, in Prof. HAMILTON'S work on *Fractures and Dislocations*, page 420.)

It will be noticed, from a slight examination of the accompanying cut, that this splint is so constructed as to entirely do away with the perineal band, or even a semblance of it, and, at the same time, affording a secure, perfect, and reliable counter-extension, without any of the objections so strongly and justly urged against all other methods, such as excoriation and ulceration of the perineum and axilla, pressure upon the obturator, crural, and axillary nerves, loosening of dressings by the patient, etc., etc. The apparatus also combines all the advantage gained by the weight and pully, as well as the fixed dressing of DESAULT.

I think that this splint entirely and satisfactorily fills a desideratum long admitted to exist in the treatment of fractured femurs, no matter in what part of the bone occurring. Also, that shortening, and all other deformities heretofore resulting from the most carefully and judiciously applied apparatus, is fully obviated by the instrument now invented, which completely overcomes all the difficulties encountered in the treatment of these fractures.

The following description of the cut will aid the surgeon in constructing and applying the splint:—

The steel spring A, passing from the small bar C, to the point of the shoulder, should be made in such a manner as to have all the spring between the centre of the bow and the hook, and of such strength as to yield from one-half to three-fourths of an inch on the application of from three to five pounds weight.

By the use of this spring we get constant and steady traction upon the muscles, which overcomes their contractility, and the limb is kept extended to its proper length, without any trouble, and with but very little traction.

The main bar F, is three inches wide, five-eighths of an inch in thickness, and twenty-eight inches long, either end of which is grooved for the reception of the two smaller bars C and E, which can be adjusted by means of the set screw C, and the extending screw E, so that the apparatus may be used for the longest or shortest patient. The two smaller bars are one and a-half inches wide, and three-eighths of an inch thick. The extending bar E, is eighteen inches long, and the counter-extending bar C, is fourteen inches long.

To the inferior extremity of the extending bar E, is attached a cross-bar, to which is attached a foot piece, and a portion of the screw-extending apparatus. By the means of the thumb-screw E, extension may be made at any time, without removing any of the dressings or disturbing the limb in the least. The oblique counter-extending adhesive plaster H, commences at the middle of the crest of the ilium, and extends obliquely across the chest anteriorly to the shoulder, then obliquely downwards and backwards to the point of commencement, (the middle of the crest of the ilium.)

The cut exhibits another counter-extending adhesive plaster (which by mistake was not lettered,) that commences a little internal to the middle of the crest of the ilium of the injured side, and extends upward to the shoulder of the same side, then downward on the dorsal aspect of the chest, to a point opposite the place of commencement.

G, adhesive plaster passing quite around the body, covering the ends of the plasters H and the one not lettered, binding them firmly to the surface, which prevents their slipping when extension is applied.

The counter-extending plasters should be from one to three inches in width, according to the size and strength of the patient, or according to the amount of force necessary to be used in keeping the limb properly extended. The extension is made by adhesive plasters in the ordinary manner.

B, I, and J, are straps of webbing, which are made fast to the main bar, and are buckled around the limbs and body.

In cases of compound fractures, this apparatus may be modified, so that the wound may be dressed without removing the splint, by cutting out a section of the main bar, and riviting strong pieces of iron to each piece, which may be bent in such a manner as to leave the wound exposed.

The apparatus here described was used by Dr. TREAT and myself, in a case of oblique fracture of the femur, occurring in the lower half of the lower third of the bone.

The limb was dressed in the ordinary manner, with short splints, to keep the ends of the fractured bone in perfect opposition. The cure was perfectly satisfactory to both patient and attendants. The unfortunate member is quite as long as its more fortunate fellow, and perfectly natural in its appearance, there being scarcely any callous.

The patient was a young man, 18 years of age, and of a rheumatic diathesis. Hence he complained some of rheumatic pains during the progress of reunion of the bone, but never found the least fault with the apparatus. The extension and counter-extension being borne as well the last as the first day of their application. There was not even the slightest abrasion of the cuticle, notwithstanding the very hot weather during the period of his confinement, it being from the 13th of June to the 1st of August.

Indulging the hope that the profession will give this new method of treating fractures of the femur and leg a fair and impartial trial, I am confident that the suffering of the unfortunate ones will be infinitely diminished, and the success of the profession greatly augmented.

## Figures and Tables

**Figure f1:**